# Coronavirus-related health literacy and knowledge among German fourth-graders

**DOI:** 10.1093/eurpub/ckac131.370

**Published:** 2022-10-25

**Authors:** T Bollweg, O Okan

**Affiliations:** TUM Department of Sport and Health Sciences, Technical University Munich, Munich, Germany

## Abstract

**Background:**

Little is known about how easy it is for children to deal with coronavirus-related information, or to what degree children have accurate knowledge about the virus. However, both might influence children’s preventive behaviors. Thus, this study explores children’s coronavirus-related health literacy (CHL) and related knowledge.

**Methods:**

In a classroom survey among fourth graders’ in North-Rhine Westphalia, Germany (07/21 - 11/21), 4 items were used to assess CHL. 5 items were used to measure coronavirus knowledge, while also assessing the frequency of speaking about the coronavirus with parents. Sociodemographic characteristics were recorded, including family affluence (FAS). Bivariate correlations are reported.

**Results:**

n = 364 students are included (49.5% female), the mean age is 9.5 years (SD=.69). 47.5% of the sample find it rather difficult or very difficult to find information about the coronavirus, while this share is 36.1% for understanding, and 39.1% for appraising coronavirus information. 22.4% say it’s difficult to not infect themselves or others (applying information). Dealing with coronavirus-related information is more difficult for children who less frequently talk to their parents about the virus (ρ = 0.166, p<.01) and have lower family affluence (r=.119, p<.05). While certain misbeliefs are rare (“the coronavirus doesn't exist”; 3.9% agreement; “Children can't get infected”; 3.0%), the belief that eating healthy prevents an infection (15.6%), that all infected people have coughs and fever (62.8%), and that a face mask doesn't help prevent an infection (44.3%) are more prevalent. Children who have a home language other than German state more inaccurate knowledge (p<.05).

**Conclusions:**

This study suggests that children’s needs for accessible and understandable information on the coronavirus were met only party. Also, certain misbeliefs are prevalent which might undermine adherence to preventive measures. More research is needed to verify these findings.

**Key messages:**

• In our sample, children’s needs for accessible and understandable information on the coronavirus were met only party.

• Misbeliefs about the coronavirus are prevalent which might undermine preventive measures.

